# Identification of Synaptic DGKθ Interactors That Stimulate DGKθ Activity

**DOI:** 10.3389/fnsyn.2022.855673

**Published:** 2022-04-27

**Authors:** Casey N. Barber, Hana L. Goldschmidt, Qianqian Ma, Lauren R. Devine, Robert N. Cole, Richard L. Huganir, Daniel M. Raben

**Affiliations:** ^1^Department of Biological Chemistry, Johns Hopkins University School of Medicine, Baltimore, MD, United States; ^2^Solomon H. Snyder Department of Neuroscience, Johns Hopkins University School of Medicine, Baltimore, MD, United States

**Keywords:** neurotransmission, synapse, lipids, synaptic vesicles, Syt1, APEX2, proximity labeling

## Abstract

Lipids and their metabolic enzymes are a critical point of regulation for the membrane curvature required to induce membrane fusion during synaptic vesicle recycling. One such enzyme is diacylglycerol kinase θ (DGKθ), which produces phosphatidic acid (PtdOH) that generates negative membrane curvature. Synapses lacking DGKθ have significantly slower rates of endocytosis, implicating DGKθ as an endocytic regulator. Importantly, DGKθ kinase activity is required for this function. However, protein regulators of DGKθ’s kinase activity in neurons have never been identified. In this study, we employed APEX2 proximity labeling and mass spectrometry to identify endogenous interactors of DGKθ in neurons and assayed their ability to modulate its kinase activity. Seven endogenous DGKθ interactors were identified and notably, synaptotagmin-1 (Syt1) increased DGKθ kinase activity 10-fold. This study is the first to validate endogenous DGKθ interactors at the mammalian synapse and suggests a coordinated role between DGKθ-produced PtdOH and Syt1 in synaptic vesicle recycling.

## Introduction

Neurotransmission occurs at each of the brain’s nearly 100 trillion synapses and governs every aspect of human life from movement to emotional processing to learning and memory (Schweizer and Ryan, 2006; Eroglu and Barres, 2010). Because dysregulation of neurotransmission can, therefore, result in a variety of neurological dysfunctions, the synaptic vesicle cycle must be tightly coordinated by a variety of proteins and enzymes (Südhof, 1995; Puchkov and Haucke, 2013).

Recently, lipids have emerged as a new critical point of regulation of neurotransmission, such as phosphatidic acid (PtdOH) because of its roles in many cellular signaling pathways and membrane rearrangements (Rohrbough and Broadie, 2005; Thakur et al., 2019; Tanguy et al., 2021). Because PtdOH is a cone-shaped lipid, it induces negative membrane curvature necessary for synaptic vesicle fusion (McMahon and Boucrot, 2015; Tanguy et al., 2019). PtdOH is also negatively charged and has a relatively small phosphomonoester headgroup, making it a popular binding partner for many proteins such as the plasma membrane-associated SNARE protein syntaxin-1 and N-ethylmaleimide sensitive fusion protein (NSF) (Zhukovsky et al., 2019). A mutation in syntaxin-1 that prevents PtdOH-binding results in a significant effect on evoked exocytosis in chromaffin and PC12 cells (Thakur et al., 2019; Zhukovsky et al., 2019; Tanguy et al., 2020). *In vitro*, PtdOH regulates the membrane insertion and activity of dynamin-1 (Burger et al., 2000). It is hypothesized that the local production of PtdOH in the active zone or periactive zone by PtdOH-producing lipid-metabolizing enzymes serves as an additional step of regulation to synaptic vesicle fusion and fission (Südhof, 1995; Rohrbough and Broadie, 2005).

Diacylglycerol kinase theta (DGKθ) is a lipid-metabolizing enzyme that phosphorylates diacylglycerol to produce PtdOH (Goto and Kondo, 1999; Tu-Sekine and Raben, 2011; Tu-Sekine et al., 2016; Sakane et al., 2020). There are 10 mammalian DGK isoforms (α, β, γ, δ, η, κ, ε, ζ, ι, θ) expressed in the central nervous system (CNS), which are categorized according to their domain structure (Barber and Raben, 2020). DGKθ is a Type V DGK, containing three C1 domains, a central PH domain, followed by the catalytic domain. Multiple DGKs have known functions within the CNS (Topham and Prescott, 2009). For example, DGKα is selectively expressed in oligodendrocytes and is thought to be involved in the production of myelin (Goto and Kondo, 1999). DGKβ is expressed postsynaptically in pyramidal excitatory neurons and evidence suggests a functional role in long-term potentiation. DGKs δ and η are thought to have roles in neurodegenerative and psychiatric diseases, such as epilepsy and bipolar disease, respectively (Caricasole et al., 2002; Baum et al., 2008). Additionally, DGKζ contains a PDZ domain, is known to bind to the postsynaptic scaffolding protein PSD-95 and data suggests a role for DGKζ in dendritic spine maintenance (Kim et al., 2009).

DGKθ is predominantly expressed in excitatory neurons throughout all parts of the mammalian brain (Ishisaka and Hara, 2014). It is expressed throughout the cytosol both pre- and postsynaptically (Goldschmidt et al., 2016; Barber and Raben, 2020). Importantly, it has been shown that DGKθ knockout (KO) neurons had significantly slower rates of synaptic vesicle endocytosis in response to depolarization as compared to wild-type (WT) neurons. The rates further slowed in response to stronger stimulation. Interestingly, rates of exocytosis were unaffected in DGKθ KO neurons compared to WT neurons (Goldschmidt et al., 2016). This suggests that DGKθ kinase activity regulates synaptic vesicle endocytosis. The mechanism underlying the regulation of this enzyme in neurons is unclear. Previously, *in vitro* studies with purified DGKθ revealed that proteins with polybasic regions could activate the enzyme (Tu-Sekine and Raben, 2012), but identification of endogenous protein activators of DGKθ in neurons has never been explored.

In the current study, we used proximity labeling and mass spectrometry to identify endogenous regulators of DGKθ’s kinase activity using an APEX2-tagged DGKθ in primary cortical neuron cultures, with an emphasis on synaptic bouton or vesicle proteins. The potential interactions were confirmed biochemically and each interactor was examined for its ability to stimulate DGKθ activity *in vitro*. We found that several well-known protein regulators of the synaptic vesicle cycle interact with DGKθ, and that some of these proteins also stimulate DGKθ activity, such as synaptogyrin-1 (Syngr1) and CaMKIIα. Importantly, synaptotagmin-1 (Syt1) interacts with DGKθ and increases DGKθ activity 10-fold. Taken together, these results suggest that DGKθ interacts with and is modulated by key synaptic vesicle recycling proteins to regulate the dynamics of synaptic vesicle recycling.

## Materials and Methods

### Reagents

**Table:** 

Primary Antibodies	Source	Product Number	IF Dilution	WB Dilution
Chicken anti-GFP	Abcam	ab13970	1:1000	–
Mouse anti-DGKθ	BD Biosciences	610931	1:100	1:100
Guinea pig anti-vGlut1	Synaptic Systems	135304	1:2500	–
Rabbit anti-DsRed	Clontech	632496	1:1000	–
Mouse anti-GFP N86/8	NeuroMab	75-131	–	1:1000
Mouse anti-tubulin	Sigma-Aldrich	T8328	–	1:1000
Rabbit anti-dynamin1	Synaptic Systems	115003	–	1:1000
Rabbit anti-synaptogyrin1	Synaptic Systems	103003	–	1:1000
Rabbit anti-munc18-1	Cell Signaling Technology	13414P	–	1:1000
Rabbit anti-munc18-1	Synaptic Systems	116003	–	1:1000
Mouse anti-clathrin heavy chain1	BD Biosciences	610500	–	1:2000
Rabbit anti-synaptotagmin1	Cell Signaling Technology	14558S	–	1:1000
Rabbit anti-camk2a	Cell Signaling Technology	D10C11	–	1:1000
Rabbit anti-hspa8	Aviva Systems Biology	ARP48445_P050	–	1:1000
Rabbit anti-syntaxin7	Synaptic Systems	110073	–	1:1000
Mouse anti-synaptojanin1	BD Biosciences	612248	–	1:1000
Rabbit anti-SNAP25	Cell Signaling Technology	3926S	–	1:1000
Rabbit anti-PKCa	Cell Signaling Technology	59754S	–	1:5000
Rabbit anti-basp1	Aviva Systems Biology	ARP59932_P050	–	1:1000
Rabbit anti-cofilin1	Cell Signaling Technology	5175T	–	1:1000
Mouse anti-HA ascites	Homemade	N/A	–	1:2500
Mouse anti-myc ascites	Homemade	N/A	–	1:2000
Rabbit anti-clathrin heavy chain1	Cell Signaling Technology	4796T	–	1:1000
Rabbit anti-myc serum	Homemade	N/A	–	1:5000

**Secondary Antibodies**	**Source**	**Product Number**		

IRDye 800CW goat anti-rabbit IgG	LICOR	926-32211	–	1:10,000
IRDye 800CW goat anti-mouse IgG	LICOR	926-32210	–	1:10,000
IRDye 680LT goat anti-rabbit IgG	LICOR	926-68021	–	1:10,000
IRDye 680LT goat anti-mouse IgG	LICOR	926-68020	–	1:10,000
IRDye 680LT streptavidin	LICOR	926-68031	–	1:10,000
IRDye 800CW streptavidin	LICOR	926-32230	–	1:10,000
Goat anti-Guinea Pig Alexa Fluor 647	Invitrogen	A21450	1:500	–
Goat anti-Mouse Alexa Fluor 647	Invitrogen	A21236	1:500	–
Goat anti-Chicken DyLight 488	Invitrogen	SA5-10070	1:500	–
Goat anti-Mouse Alexa Fluor 568	Invitrogen	A11004	1:500	–
Goat anti-Rabbit Alexa Fluor 568	Invitrogen	A11036	1:500	–

**Purified Proteins**	**Source**	**Product Number**		

Camk2a	Origene	TP318186		
Hspa8	Origene	TP302209		
Synaptogyrin-1	Origene	TP300562		
Dynamin-1	Origene	TP306284		
Munc18-1	Origene	TP318471		
Syntaxin-7	NovoPro Bioscience	502965-20		

**Other Reagents**	**Source**	**Product Number**		

Biotinyl tyramide	Chemodex	B0270		
Trypsin	Worthington	LS003741		
Streptavidin beads	Pierce	88817		
Hydrogen peroxide	Sigma-Aldrich	H1009-100ML		
1,1,1,3,3,3-Hexafluoro-2-propanol	Acros Organics	AC445820100		
Sodium ascorbate	Spectrum	S1349		

**Table:** 

**Primary Antibodies**	**Source**	**Product Number**
Sodium azide	Thermo Fisher Scientific	14314
Trolox	Acros Organics	53188-07-1
Iodoacetamide	Sigma-Aldrich	I6125-25G
DTT	Pierce	A39255
Biotin	Sigma-Aldrich	B4501-1G
Lipofectamine 2000	Invitrogen	11668-019
		

### Plasmids

**Table:** 

Plasmid	Source	Product Number
pRK5-myc-DGKθ	Homemade	N/A
pCAG_DGKθ-GFP-APEX2	Homemade	N/A
LCK-myr-mCherry	Homemade	N/A
pcDNA3.1 HA-dynamin1	Addgene	34682
pCAG-mGFP-Camk2a	Addgene	127389
pcDNA3.1 GFP-HSPA8	Addgene	121161
pCAG-Syt1-pH	Homemade	N/A
pCMV5-HA-Stx7	MRC PPU Reagents and Services	26785
pCMV5-HA-CLTC	MRC PPU Reagents and Services	31877
pCMV5-Syngr1-GFP	Provided by Raffaella de Pace, NIH/NICHD	N/A
pCMV5-HA-Munc18-1b	Provided by Jacqueline Burré, Cornell	N/A
pCMV5-Munc18-1b-GFP	Provided by Jacqueline Burré, Cornell	N/A
		

### Neuron Culture

All animal protocols were approved by the Johns Hopkins University Animal Care and Use Committee. To prepare primary neuron cultures, WT embryonic day 16 (E16) rat pups were removed from sacrificed Sprague Dawley mothers. Cortices were dissected from pup brains and incubated in dissection media with papain and DNase for 25 min at 37°C and then fully dissociated by gentle trituration. Neurons to be used for APEX2 labeling were then electroporated with the Rat Nucleofector Kit (Amaxa) with 4 μg pCAG_DGKθ-GFP-APEX2 and plated on poly-L-lysine (PLL)-coated 10 cm dishes in Neurobasal Plus medium supplemented with 5% horse serum, 50 U/mL penicillin, 50 U/mL streptomycin, 2 mM Glutamax and 2% b27 Plus at 10 e^6^ cells/dish. WT neurons were plated in the same media at 4 e^6^ cells/dish. At DIV4, all neurons were fed with serum-reduced (2% horse serum) media supplemented with fluoro-deoxyuridine (FDU) to prevent the proliferation of glial cells. Following this, neurons were fed about every 4 days with Neurobasal Plus medium supplemented with 50 U/mL penicillin, 50 U/mL streptomycin, 2 mM Glutamax, and 2% b27 plus. For most experiments, neurons were grown until DIV21 in a 37°C incubator (5%CO_2_).

### Neuron Immunofluorescence

For immunofluorescence experiments, WT rat neurons were plated on PLL-coated 18 mm glass coverslips placed in the wells of a 12 well plate. On DIV19, neurons were transfected with a combination of 0.5 μg pCAG_DGKθ-GFP-APEX2, 0.5 μg LCK-myr-mCherry, or 0.5 μg pRK5-myc-DGKθ. To accomplish this, Lipofectamine 2000 (L2K) was diluted in Neurobasal media and incubated for 5 min. DNA was diluted in Neurobasal media and then combined at a 1:1 ratio with the L2K/Neurobasal dilution and incubated for 15 min at room temperature (RT). 1 mL of media was gently removed from each well of cells to be transfected and saved. The transfection mixture was then added dropwise to cells and incubated for about 13 min at 37°C, 5%CO_2_. Following this incubation, the transfection media was aspirated and replaced with 1 mL of the saved media. The next day, the neurons were fixed for staining. Coverslips were washed 1× with phosphate-buffered saline (PBS) and then incubated with parafix (4% paraformaldehyde + 4% sucrose in PBS) for 15 min at RT and washed 3× with PBS. They were permeabilized for 10 min with 0.25% Triton-X in PBS and washed 3× with PBS. To reduce non-specific antibody binding, coverslips were incubated with 10% bovine serum albumin (BSA) at 37°C (5%CO_2_) for 1 h. Coverslips were then incubated with primary antibodies diluted in 3% BSA overnight at 4°C. The next day, coverslips were washed 3× with PBS and incubated with secondary antibodies (goat conjugated Alexa Fluor 647, 568, or 488) diluted in 3% BSA in PBS +0.1% Triton-X (PBST) for 1 h at RT. Subsequently, they were washed 3× with PBS and mounted on glass slides with Fluoromount-G and stored at 4°C. Images were obtained with an 800-laser scanning confocal microscope (Zeiss) and images were analyzed with ImageJ.

### Western Blot Analysis

All SDS-PAGE gels were performed with Bolt 8% Bis-Tris mini protein gels. Eluates from streptavidin pulldowns and CoIPs were loaded directly on gels. Input samples and other neuronal or HEK lysate samples were diluted with water and 4× Laemmli buffer with 10% β-mercaptoethanol and boiled for 5 min at 100°C before loading on gels. Proteins were transferred to nitrocellulose membranes with the Bio-Rad Mini *Trans-*Blot system and blocked with 3% BSA for 1 h. Blots were incubated with primary antibodies diluted in 3% BSA in PBST on a 4°C rocker overnight. The next day, blots were washed 3× with PBST and incubated with LICOR IRDye-conjugated secondary antibodies for 1 h at RT. Afterward blots were washed 3× with PBST before infrared imaging with an Odyssey Imaging System.

### APEX2 Proximity Labeling

At DIV21, neurons expressing pCAG_DGKθ-GFP-APEX2 were incubated with aCSF supplemented with 500 μM biotin-phenol for 30 min at 37°C. Control neurons not incubated with biotin-phenol were incubated with non-supplemented aCSF for 30 min at 37°C. Immediately after incubation, aCSF was aspirated and 10 mL of 50 mM KCl in aCSF was added to each dish to induce depolarization for 30 s (non-depolarized neurons did not receive this treatment). Following depolarization, 1 mM H_2_O_2_ was added to each dish of cells for 1 min to catalyze the APEX2 labeling reaction. After 1 min, aCSF containing biotin-phenol and H_2_O_2_ was aspirated and quencher solution (10 mM sodium ascorbate, 5 mM Trolox, 10 mM sodium azide in DPBS) was added to each dish of cells to quench the labeling reaction. Neurons were washed with this solution 4×. Following the last wash, neurons were harvested in 1 mL RIPA containing protease inhibitors, each of the quenchers, and 50 mM TEABC. Each lysate was incubated on a rotator at 4°C for 20 min and then centrifuged at 17,000 × *g* for 5 min. The supernatant was transferred to a new tube and placed at −20°C until preparation for proteomic analysis.

### Mass Spectrometry Sample Preparation

Neurons (∼50 e^6^ cells total) from five 10 cm dishes were lysed in RIPA buffer. Proteins in lysates were reduced in 10 mM DTT at 56°C for 30 min and alkylated with 30 mM IAA at RT in the dark for 30 min prior to proteolysis with 1:20 dilution of 1 mg/mL trypsin (Worthington): protein in 50 mM TEABC buffer at 37°C on a shaker overnight. Following trypsin digestion, peptides were precipitated and acidified by adding 20% TFA in dH_2_0 to pH4 and incubated at RT for 10 min. Subsequently, lysates were spun at 10,000 × *g* for 10 min at RT to pellet debris, undigested proteins, and membranes. The supernatant was loaded on a 30 mg Waters Oasis plate (WAT058951) to bind and desalt the peptides according to the manufacturer’s instructions. The peptides were eluted with 2× washes of 500 μL 60% trifluoroacetic acid and subsequently dried in a speed vacuum.

To prepare streptavidin beads, Pierce magnetic streptavidin beads were washed 3× with PBS. A 1:8 ratio was used (mL beads: mg protein). The dried peptides were resuspended completely in 1 mL RIPA and combined with beads. The peptide-bead mixture was incubated overnight on a 4°C rotator. The next day, the beads were washed 4× with 1 mL 1% SDS, 5× 1 mL 5% acetonitrile in dH_2_0, and 3× 1 mL dH_2_0. Washes were performed by pipetting with low retention tips and after each wash, samples were transferred to a new Eppendorf tube. After the last wash, biotinylated peptides were eluted from streptavidin beads with neat hexafluoroisopropanol (HFIP). For each sample, 600 μL HFIP was added to the tube, incubated for 5 min at RT, then placed on a magnetic rack and the liquid was pipetted off and transferred to a new tube. This was done a second time and the supernatants were combined into one tube for each sample and then dried in a speed vacuum. The dried peptides were kept in −20°C until mass spectrometry analysis.

### Mass Spectrometry

Peptides were resuspended in 200 μL of 0.1% trifluoroacetic acid loaded and desalted on a Waters Oasis plate C18. After desalting with 0.1%TFA, peptides were eluted in basic (10 mM TEAB) in steps at 5, 10, 25, and 75% acetonitrile. Fractions were dried and rehydrated with 2% acetonitrile, 0.1% formic acid. Each peptide fraction was resuspended in 20 μL loading buffer (2% acetonitrile in 0.1% formic acid) and analyzed by reverse phase liquid chromatography interfaced with tandem mass spectrometry (LC/MSMS) using an Easy-LC 1200 HPLC system^1^ interfaced with an Orbitrap Fusion Lumos Tribrid Mass Spectrometer (Lumos, see text footnote 1). Peptides (20% each fraction) were loaded onto a C18 trap (S-10 μM, 120 Å, 75 μm × 2 cm, YMC, Japan) and subsequently separated on an in-house packed PicoFrit column (75 μm × 200 mm, 15 μ, +/−1 μm tip, New Objective) with C18 phase (ReproSil-Pur C18-AQ, 3 μm, 120 Å^2^) using 2–90% acetonitrile gradient at 300 nl/min over 120 min. Eluting peptides were sprayed at 2.0 kV directly into the Lumos. Survey scans (full MS) were acquired from 350 to 1,400 m/z with data dependent monitoring with a 3 s cycle time. Each precursor individually isolated in a 1.2 Da window and fragmented using HCD activation collision energy 30 and 15 s dynamic exclusion, first mass being 120 m/z. Precursor and the fragment ions were analyzed at resolutions 120,000 and 50,000, respectively, with automatic gain control (AGC) target values at 4 e^5^ with 50 ms maximum injection time (IT) and 1 e^5^ with 54 ms maximum IT, respectively.

Isotopically resolved masses in precursor (MS) and fragmentation (MS/MS) spectra were processed in Proteome Discoverer (PD) software (v2.4, Thermo Scientific). All data were searched using Mascot (2.6.2^3^) against a custom database (191113 entries, including custom sequence with DGKθ-GFP-APEX2 protein plus Rattus) using trypsin as the enzyme, 2 missed cleavages, precursor and fragment tolerances or 6 ppm and 0.01 Da, respectively. Variable modifications included Asn and Gln deamidation, Met oxidation, and Cys carbamidomethylation as static modification. Target decoy PSM (peptide spectrum match) validator was used, and any low-scoring spectra were sent to a custom BYONIC search against SwissProt_Rat_032019_8060entries_DGK_Apex database. The enzyme was specified as trypsin, with 2 missed cleavages, precursor and fragment tolerances or 6 and 8 ppm, respectively. Total common modifications 2 max, rare modifications 1 max. Fixed modification of Carbamidomethyl / +57.021464 @ C, Dynamic Modifications of Oxidation / +15.994915 @ M | common1, Deamidated / +0.984016 @ N, Q | common1, Carbamyl / +43.005814 @ K, R | rare1, Acetyl / +42.010565 @ NTerm, Protein NTerm, K | common2, Acetyl / +42.010565 @ NTerm, Protein NTerm, K | common2, Phospho / +79.966331 @ S, T, Y | rare1, Trioxidation / +47.984744 @ W | rare1, Gln- > pyro-Glu / −17.026549 @ NTerm Q | common2, Formyl / +27.994915 @ NTerm, K, S, T | rare1, Dimethyl / +28.0313 @ K, R | rare1, Biotin Tyramide / +361.1460 @ Y | rare1, Biotin Tyramide_Plus1 / +362.149 @ Y | rare1.

Spectra and peptides with high confidence (1% FDR) were used for feature mapping and alignment, RT up to 10 min, Mass tolerance 10 ppm, min S/N threshold 5. Quantification calculated from precursor intensity, using unique peptides only. No scaling or normalization, protein abundance calculated from summed abundances, top 3, pair wise ratio based. Hypothesis test was set to *t*-test (background based).

### Streptavidin Pulldowns

For streptavidin pulldowns to confirm biotinylation of proteins, APEX2 labeled neuronal lysates were combined with magnetic streptavidin beads that had been washed 2× with RIPA. Following addition of the lysate to the beads, RIPA was added to the lysate-bead mixture to facilitate mixing and samples were incubated overnight on a 4°C rotator. The following day, the flow-through was pipetted off the magnetic beads and the beads were washed 2× 1 mL RIPA, 1× 1 mL 1 M KCl, 1× 1 mL 0.1 M Na_2_CO_3_, 1× 1 mL 2 M urea in 10 mM Tris–HCl pH8, 2× 1 mL RIPA. To elute biotinylated proteins from the beads, Laemmli sample buffer containing 6 mM biotin and 60 mM dithiothreitol (DTT) was added to the beads and incubated at 100°C for 15 min. Afterward, the supernatant was collected from the magnetic beads and loaded on gel for Western blot analysis.

### Coimmunoprecipitation

All CoIPs were performed using HEK293FT cells. Cells were seeded on 10 cm dishes in DMEM supplemented with 10% fetal bovine serum and 1% Pen-Strep. Transfection was performed when the cells reached about 80% confluency. Lipofectamine 2000 (L2K) was diluted in Opti-MEM and incubated for 5 min at RT. 4 μg of pRK5-myc-DGKθ alone or 4 μg pRK5-myc-DGKθ plus 4 μg of each potential interactor DNA were diluted in Opti-MEM. The DNA and L2K were mixed at a 1:1 ratio and incubated for 15 min at RT. Afterward, the DNA/L2K mixture was added to cells dropwise and the cells were placed in the incubator (37°C, 5% CO_2_) for 2 h. Then, the media containing the transfection mixture was aspirated completely from plates and fresh HEK cell media was replaced. The cells were harvested after 2 days. Before lysis, cells were washed 2× with PBS. After the final wash was aspirated, the cells were lysed in 1 mL CoIP buffer (1% NP40, 5 mM NaF, 5 mM NaPPi, 1 mM EDTA, 1 mM EGTA in 1× PBS) and placed on 4°C rotator for 20 min. The lysates were then centrifuged at 17,000 × *g* for 5 min and the supernatant was transferred to a new tube.

To begin CoIPs, lysates were first precleared by incubating with protein A/G beads for 1 h on 4°C rotator to reduce non-specific binding. After incubation, samples were centrifuged at 10,000 × *g* and the supernatant was collected and transferred to a new tube. Subsequently, 2 μL of rabbit anti-myc or mouse anti-myc ascites antibodies were added to each sample and incubated overnight on 4°C rotator. The next day, protein A/G beads were added to each antibody/lysate mixture and incubated for 2 h on RT rotator. The flow-through was then removed and beads were gently washed 3× with CoIP buffer by pipetting. After the final wash, beads were incubated in elution buffer (50% CoIP buffer, 50% 4× Laemmli sample buffer) at RT for 15 min and then at 100°C for 15 min. Samples were then briefly centrifuged and the supernatant was transferred to a new tube. Samples were kept at −20°C until samples were analyzed for the presence of the interactor by Western blot analyses.

### DGKθ Activation Assay

The ADP-Glo Assay (Promega) was used to examine the ability of each DGKθ interactor to stimulate DGKθ activity (Balzano et al., 2011; Nagaraj et al., 2017). This assay kit employs a fluorescent reagent to quantify the amount of ADP generated by DGKθ’s kinase reaction. The reaction mix consists of 10 ng/μL purified DGKθ, 0.5 mM of the water-soluble DAG substrate 1,2-dihexanoyl-sn-glycerol (Dic6), 1 mM ATP, 3 mM MgCl_2_, and 500 ng/μL of the potential activator. The reaction mixture was incubated at 37°C for 10 min and stopped by the addition of an equal volume of the ADP-Glo Reagent. This was incubated at RT for an additional 40 min to deplete the remaining ATP. Kinase Detection Reagent was then added and incubated at RT for 40 min, which converts the kinase-generated ADP to ATP which is quantified using a luciferase/luciferin-based reaction. The resulting luminescence is then measured with a Molecular Devices SpectraMax^®^ i3x Multi-Mode Microplate Reader.

## Results

### Validation of DGKθ-GFP-APEX2 Expression and Activity

To gain insight into the mechanism of DGKθ’s modulatory role in compensatory endocytosis, we performed APEX2 proximity labeling with APEX2-tagged DGKθ in cortical rat neurons. Mass spectrometry analysis was used to identify potential interactors, with a particular focus on proteins with a known role in synaptic vesicle recycling. We chose to employ APEX2 proximity labeling because its rapid labeling of proteins (∼1 min) within a small radius (∼10 nm) results in specific identification of interactions and has been previously used in multiple similar contexts (Rhee et al., 2013; Hung et al., 2014, 2016; Lam et al., 2015; Loh et al., 2016; Chung et al., 2017; Han et al., 2017; Martell et al., 2017). To identify specific, functionally relevant DGKθ interactors, we depolarized neurons prior to labeling in live cells for some samples.

**FIGURE 1:**
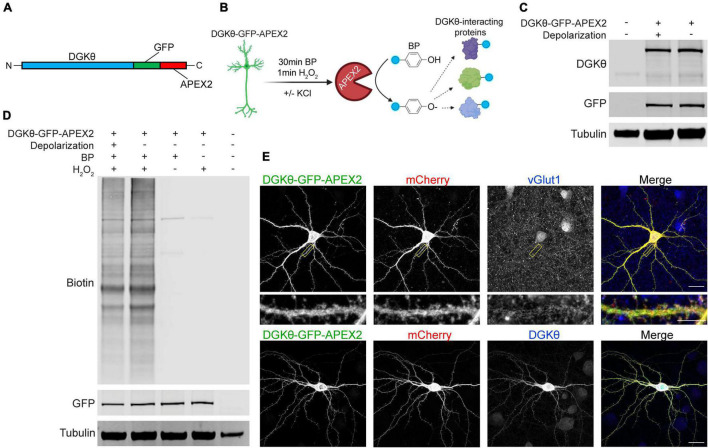
DGKθ-GFP-APEX2 is expressed ubiquitously throughout neurons and labeling of interactors is dependent on H_2_O_2_. **(A)** DGKθ-GFP-APEX2 was cloned into the neuronal expression vector pCAG. **(B)** Cortical neurons (DIV21) expressing DGKθ-GFP-APEX2 were incubated with biotin-phenol (BP, 500 μM, 30 min). Depolarized neurons were treated with a 30 s incubation with 50 mM KCl; control neurons were treated with aCSF. The addition of H_2_O_2_ (1 mM, 1 min) catalyzes the reaction. Figure made with BioRender.com. **(C)** DIV21 cortical neuron lysates express near equal amounts of DGKθ-GFP-APEX2 with and without depolarization. **(D)** DGKθ-GFP-APEX2 biotinylated proteins with similar efficacy in depolarized and non-depolarized samples, and labeling is dependent on BP and H_2_O_2_. **(E)** In cortical neurons (DIV19), DGKθ-GFP-APEX2 is localized throughout the cell, in spines, and colocalizes with vGlut1 and DGKθ overexpression. Scale bars represent 20 and 5 μm (cropped region).

We first confirmed the expression, localization, and activity of our DGKθ-GFP-APEX2 construct (Figure 1A). Mature cortical neurons expressing DGKθ-GFP-APEX2 were incubated with biotin-phenol (BP) for 30 min, followed by a 1 min incubation with H_2_O_2_ to catalyze the labeling reaction. This was performed with or without KCl to induce depolarization (see section “Materials and Methods”) (Figure 1B). The DGKθ-GFP-APEX2 construct expresses well in neurons in both depolarized and non-depolarized neurons, as evidenced by both a GFP and DGKθ antibody. The DGKθ antibody recognizes the construct at 150 kDa and endogenous DGKθ at 100 kDa (Figure 1C). We then tested the BP labeling efficiency of the DGKθ-GFP-APEX2 construct in live neurons. The resulting Western blots showed that APEX2 labeling of DGKθ-interacting proteins is dependent on BP and H_2_O_2_ and there are no significant differences in labeling efficacy between the depolarized and non-depolarized samples. The detection of endogenously biotinylated proteins is seen in the lysates from control reactions, with BP or H_2_O_2_ omitted (Figure 1D).

To examine the localization of the construct, mature neuron cultures were transfected with DGKθ, DGKθ-GFP-APEX2, or mCherry. Neurons were fixed and stained with antibodies against DGKθ, GFP, vGlut1, or DsRed. As expected, DGKθ-GFP-APEX2 is expressed cytosolically throughout the cell, as evidenced by near-complete colocalization with DGKθ and the mCherry cell-fill (Figure 1E, lower panel). DGKθ-GFP-APEX2 is also expressed synaptically, illustrated by GFP signal within spines and colocalization with vGlut1 expression (Figure 1E, upper panel). Altogether, these results show that the DGKθ-GFP-APEX2 construct expresses well in neurons, co-localizes with overexpressed DGKθ, robustly labels proteins, and that labeling is dependent on H_2_O_2_.

### Mass Spectrometry Identification of DGKθ-GFP-APEX2 Labeled Proteins

To capture changes in DGKθ interactions with neuronal activity, we labeled mature neurons expressing DGKθ-GFP-APEX2 with and without depolarization. The selection of potential interactors was based on interaction changes dependent on depolarization. To identify DGKθ interactors, we conducted three mass spectrometry experiments with the same conditions. The detailed data from each mass spectrometry experiment can be found in Supplementary Tables 1–3 and Supplementary Figure 1. This data is also publicly available in the Proteomics Identifications Database (PRIDE) at http://www.ebi.ac.uk/pride/archive/projects/PXD030495.

**FIGURE 2:**
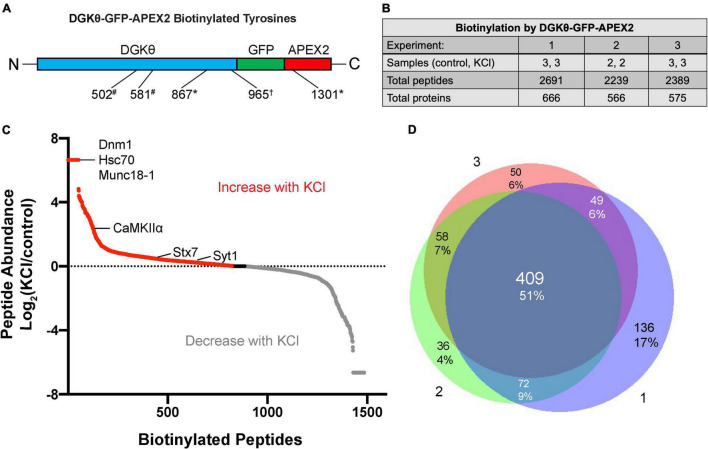
Differential labeling of synaptic proteins by DGKθ-GFP-APEX2 with KCl. **(A)** Biotinylated tyrosine residues within DGKθ-GFP-APEX2, corresponding to peptides identified in one (*), two (#), or three (†) experiments. **(B)** Mass spectrometry experiments 1 and 3 consisted of three KCl-depolarized samples and three control samples, while experiment 2 consisted of two of each. Each sample contains ∼50 e^6^ cells. The three experiments produced similar numbers of total biotinylated peptides and their corresponding proteins. **(C)** The differential labeling of peptides with KCl is illustrated for mass spectrometry experiment 2. All biotinylated peptides identified are graphed by their log_2_(peptide abundance). Peptide abundance is calculated as (relative abundance in KCl-depolarized samples)/(relative abundance in control samples). **(D)** Of the total number of peptides identified from our three mass spectrometry experiments, 51% were found in all three experiments, 22% were found in two of the three experiments, and 27% were found in only one experiment.

Because proteins were trypsinized before enrichment with streptavidin, the site of biotinylation is known for each peptide identified. Importantly, multiple biotinylated peptides corresponding to DGKθ-GFP-APEX2 were identified in each mass spectrometry experiment, serving as our positive control (Figure 2A). Each experiment produced similar numbers of total biotinylated peptides, ∼2,200–2,700, corresponding to ∼550–700 proteins (Figure 2B). Importantly, because we employed label-free quantitation, the relative abundance ratio for each biotinylated peptide is calculated as (abundance in KCl samples)/(abundance in control samples). Figure 2C displays the log_2_(abundance ratio) for each identified peptide in one of the three experiments, ranging from those peptides only found in KCl samples, those present in both samples in different amounts, and those peptides only found in control samples. Although some peptides may have only been found in one condition, there were usually other peptides from the same protein that were present in the other condition. Importantly, most of our analysis focused on differences in biotinylation and relative abundance between conditions. There was a high degree of similarity between the data sets from each experiment, increasing confidence in potential DGKθ interactors. A total of 51% of all peptides identified were found in all three experiments, with 17% being the largest amount of peptides only identified in 1 experiment (Figure 2D).

**FIGURE 3:**
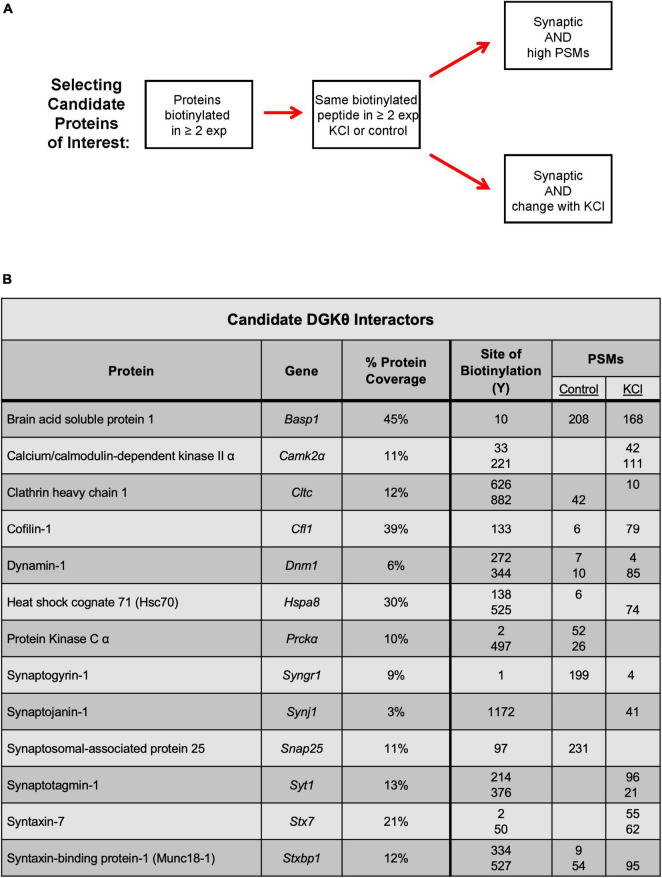
Selection of candidate DGKθ proteins of interest. **(A)** Criteria for the selection of candidate DGKθ-interacting proteins. **(B)** The selected candidate DGKθ interactors and one or two biotinylated peptides (all of which were identified in all three mass spectrometry experiments), with their PSMs in each condition. Site of biotinylation, PSMs, and % protein coverage from mass spectrometry experiment 1.

Because the goal of this project is to investigate the mechanism of DGKθ’s newly discovered regulatory role in endocytosis, we focused our analyses on proteins known to have a role in the synaptic vesicle cycle, particularly synaptic proteins. Many of those proteins had some biotinylated peptides found only within the depolarized samples, such as dynamin-1, heat shock cognate 71 (Hsc70), and syntaxin-binding protein 1 (Munc18-1) (Figure 2D). Other proteins had peptides with a high abundance ratio and a high number of PSMs (peptide-spectrum matches), such as calcium calmodulin-dependent kinase II alpha (CaMKIIα). Most of the proteins we identified as potential interactors have biotinylated peptides present in samples with and without depolarization. To select candidate proteins of interest, we first isolated proteins that were biotinylated in at least 2 experiments. Then, we selected proteins with the same biotinylated peptide in the same condition in at least 2 experiments. The final candidate proteins selected were all synaptic or directly involved in vesicle cycling. These proteins either had biotinylated peptides with a relatively high number of PSMs or a significant change in the number of PSMs or the site of biotinylation between depolarized and non-depolarized samples (Figure 3A). Our candidate DGKθ interactors consisted of the synaptic vesicle proteins listed in Figure 3B. Also listed are 1 or 2 corresponding peptides (each detected in all 3 experiments) and the number of PSMs for that peptide found in each condition in experiment 1. Supplementary Figure 1 outlines all of the biotinylated peptides and their number of PSMs for each candidate interactor from each experiment. Cofilin-1, CaMKIIα, protein kinase Cα (PKCα), Hsc70, synaptogyrin-1, SNAP-25 and synaptojanin-1 had an obvious, significant change in peptides and their number of PSMs between depolarized and non-depolarized conditions, while proteins like brain acid soluble protein 1 (Basp1) had peptides with unusually high numbers of PSMs. Clathrin heavy chain 1, dynamin-1, syntaxin-7, Syt1, and Munc18-1 had a combination of both characteristics (Figure 3B and Supplementary Figure 1).

### Biochemical Validation of Biotinylated Candidate Interactors

**FIGURE 4:**
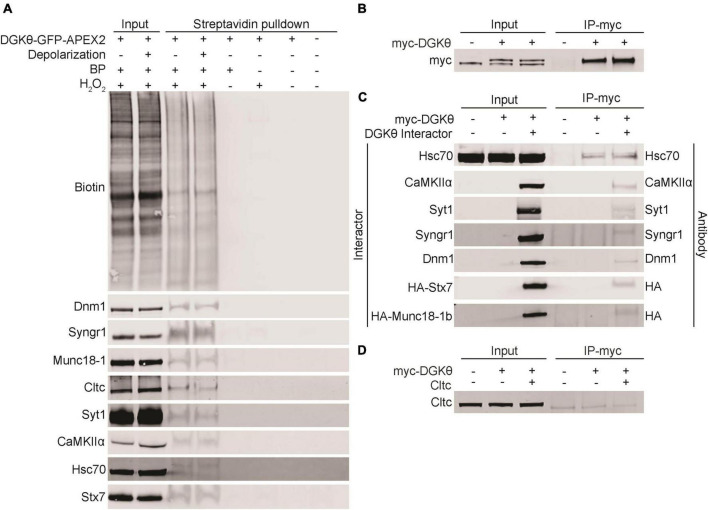
Validation of candidate DGKθ-interacting proteins. **(A)** Streptavidin beads were used to isolate biotinylated proteins from DGKθ-GFP-APEX2 labeled cortical neuron lysates (DIV21), and biotinylation was confirmed biochemically for dynamin-1, synaptogyrin-1, Munc18-1, clathrin heavy chain 1, Syt1, CaMKIIα, Hsc70, and syntaxin-7. **(B)** DGKθ overexpressed in HEK cells is immunoprecipitated with a myc antibody. **(C)** DGKθ interacts with Hsc70, CaMKIIα, Syt1, synaptogyrin-1, dynamin-1, syntaxin-7, and Munc18-1 when both are overexpressed in HEK cells. Hsc70 interacts with DGKθ at its endogenous expression level. **(D)** Clathrin heavy chain 1 does not interact with DGKθ when overexpressed in HEK cells. All blots are representative of at least three separate experiments.

Because APEX2 proximity labeling can label transient non-specific interactors, it was essential to further validate the potential interaction between DGKθ and our candidate interactors. To biochemically validate that our candidate interactors are biotinylated, we performed streptavidin pulldowns with DGKθ-GFP-APEX2-labeled lysates. A streptavidin-conjugated antibody confirmed the presence of biotinylated proteins in the input and pulldown samples from both the depolarized and non-depolarized conditions. Dynamin-1, synaptogyrin-1, Munc18-1, clathrin heavy chain 1, Syt1, CaMKIIα, Hsc70, and syntaxin-7 were detected in the eluates from both depolarized and non-depolarized samples (Figure 4A). Basp1, cofilin-1, PKCα, synaptojanin-1 and SNAP-25 were not detected in these eluates, suggesting that they are not biotinylated by DGKθ-GFP-APEX2 (Supplementary Figure 2). These data suggest that most of our candidate interactors, such as canonical synaptic vesicle recycling proteins Syt1 and Munc18-1, are biotinylated by DGKθ-GFP-APEX2, which complements our mass spectrometry results. Therefore, our list of candidate interactors now includes CaMKIIα, clathrin heavy chain 1, dynamin-1, Hsc70, synaptogyrin-1, Syt1, syntaxin-7, and Munc18-1.

### Biochemical Confirmation of the Interaction Between DGKθ and Candidate Interactors

To further confirm the interaction between DGKθ and our candidate interactors, we performed coimmunoprecipitation (CoIP) experiments in HEK293FT cells. Due to the difficulty in obtaining sufficient amounts of protein in neurons compared to HEK cell cultures, we performed these experiments in HEK cells for simplicity and increased sensitivity. HEK293FT cells were transfected with myc-DGKθ alone or together with a candidate interactor. First, we confirmed that our myc antibody efficiently pulled down DGKθ in HEK cells. Significant amounts of DGKθ were found in eluates from DGKθ-overexpressed HEK cell lysates, as visualized by a different myc antibody (Figure 4B). We found that CaMKIIα, Syt1, synaptogyrin-1, dynamin-1, syntaxin-7 and Munc18-1 coimmunoprecipitated with DGKθ when both DGKθ and the candidate interactor are overexpressed in HEK cells. Hsc70 coimmunoprecipitated with DGKθ at its own endogenous expression level as well as when overexpressed (Figure 4C). Control pulldowns were conducted using a non-specific mouse IgG and failed to pulldown any of our confirmed DGKθ interactors (Supplementary Figure 3).

We note that clathrin heavy chain 1 was biotinylated but did not coimmunoprecipitate with DGKθ (Figure 4D). This could be because the interaction is specific to neurons or that the proteins are spatially segregated in HEK cells. Further, for both Hsc70 and clathrin heavy chain 1, there was not an obvious difference in expression between endogenous and overexpression of these proteins. We hypothesize that a difference is not visible because the endogenous expression is robust.

**FIGURE 5:**
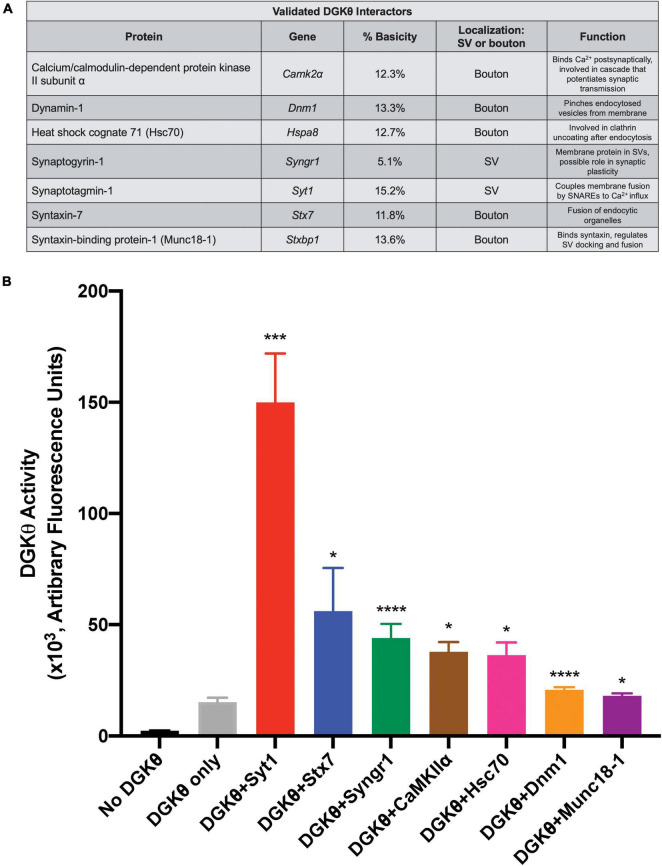
Syt1 increases DGKθ activity 10-fold over DGKθ alone. **(A)** List of candidate proteins for which an interaction with DGKθ was validated and their function. Syt1 has the highest percent basicity of the confirmed interactors. **(B)** DGKθ kinase activity was measured for DGKθ alone and for DGKθ (10 ng/μL) plus the addition of each confirmed interactor (500 ng/μL). Syt1 increased DGKθ activity 10-fold, while syntaxin-7 increased DGKθ activity four-fold. Error bars indicate SEM. Graph summarizes data from 2 experiments with three replicates each, *n* = 6, *p < 0.05, ***p < 0.0005, ****p < 0.0001 against DGKθ only, ratio paired *t*-test.

Overall, these results confirm our mass spectrometry studies, providing the first evidence that DGKθ interacts with Hsc70, CaMKIIα, Syt1, synaptogyrin-1, dynamin-1, syntaxin-7 and Munc18-1. All of DGKθ’s newly confirmed interactors have a known role in synaptic vesicle recycling, lending further support to the hypothesis that DGKθ is involved in regulating endocytosis. As we expected the DGKθ activators to contain a polybasic region, we also note that Syt1, dynamin-1 and Munc18-1 had the highest percent basicity of the proteins that coimmunoprecipitated with DGKθ (Figure 5A).

### DGKθ Interactors That Stimulate DGKθ Activity

Confirmed DGKθ interactors were examined for their ability to stimulate DGKθ’s kinase activity using the ADP-Glo assay (Balzano et al., 2011; Nagaraj et al., 2017). In this assay, kinase-generated ADP is converted to ATP which is then quantified via a luciferase/luciferin-based reaction. This reaction was performed with purified DGKθ alone (10 ng/μL) and with the addition of the following purified, recombinant proteins of each individual DGKθ interactor (500 ng/μL) validated by CoIP as described above: Hsc70, CaMKIIα, Syt1, synaptogyrin-1, dynamin-1, syntaxin-7, and Munc18-1. We found that the addition of 500 ng/μL Syt1 produced a 10-fold increase in DGKθ activity (arbitrary fluorescence units/50 ng DGKθ) over DGKθ alone, the largest increase of all our confirmed interactors (No DGKθ: 2.44 ± 0.09, DGKθ only: 15.27 ± 1.89, DGKθ+Syt1: 149.99 ± 21.91, DGKθ+Stx7: 56.11 ± 19.32, DGKθ+Syngr1: 43.95 ± 6.35, DGKθ+CaMKIIα: 37.83 ± 4.28, DGKθ+Hsc70: 36.29 ± 5.69, DGKθ+Dnm1: 20.66 ± 1.16, DGKθ+Munc18-1: 18.00 ± 1.07; ×10^3^ arbitrary fluorescence units, *n* = 6). It is also noteworthy that addition of syntaxin-7 led to a four-fold increase in DGKθ activity, while synaptogyrin-1, CaMKIIα and Hsc70 all led to a 2.5-fold increase in DGKθ activity. Dynamin-1 and Munc18-1 did not cause activation of DGKθ (Figure 5B). From this data, we conclude that Syt1 is the most probable DGKθ interactor and activator, while syntaxin-7 and synaptogyrin-1, proteins also implicated in vesicle recycling, may also play roles in DGKθ activity.

## Discussion

In previous studies, DGKθ kinase activity was shown to have a regulatory role in synaptic vesicle endocytosis, particularly during elevated neuronal activity (Goldschmidt et al., 2016). This role depends on the catalytic activity of DGKθ. The mechanism involved in regulating DGKθ activity in synaptic boutons has not been explored, but it is known that proteins with polybasic regions activate the enzyme’s activity (Tu-Sekine and Raben, 2012). The current study is the first to identify endogenous DGKθ interactors in neurons. Using APEX2 proximity labeling, mass spectrometry analyses, and biochemical analyses we confirmed that DGKθ interacts with Hsc70, CaMKIIα, Syt1, synaptogyrin-1, dynamin-1, syntaxin-7, and Munc18-1. Importantly, Syt1, syntaxin-7, synaptogyrin-1, Hsc70 and CaMKIIα all increased DGKθ kinase activity compared to DGKθ alone. Notably, Syt1 produced a 10-fold increase in DGKθ activity, suggesting Syt1 is the strongest DGKθ interactor. Taken together, these results suggest that Syt1 functions at synapses to regulate PtdOH production by DGKθ.

**FIGURE 6:**
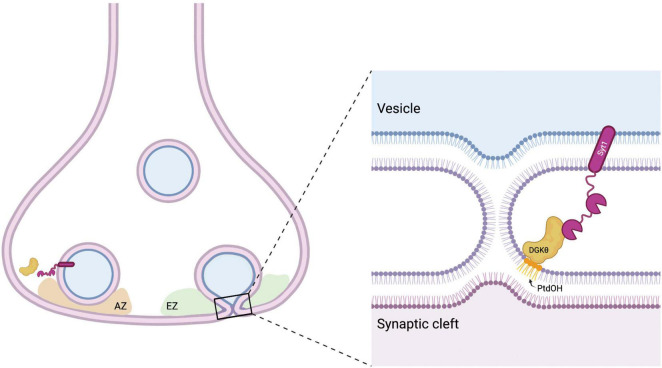
Model for DGKθ’s interaction with Syt1. Syt1 is a transmembrane protein on synaptic vesicles and the presynaptic membrane. Perhaps to aid in the membrane deformation required in the fusion and fission of vesicles, Syt1 binds to DGKθ and stimulates its kinase activity, producing local hotspots of PtdOH that generate negative membrane curvature. AZ = active zone, EZ = endocytic zone. Figure made with BioRender.com.

Given the role of polybasic regions in modulating DGKθ activity (Tu-Sekine and Raben, 2012), it is of particular note that Syt1 is the interactor with the highest percent basicity. The C2B domain of Syt1 contains a polybasic patch (K324-327) that has been shown to bind acidic phospholipids and regulates synchronous evoked synaptic vesicle release (Chang et al., 2018). The binding of Ca^2+^ to Syt1 results in the hydrophobic amino acids at the ends of the Ca^2+^-binding domains to insert into membranes. This conformational change generates membrane curvature and triggers fusion of synaptic vesicles with the presynaptic membrane (Martens et al., 2007; Lynch et al., 2008; Hui et al., 2009). Importantly, Syt1 also serves as a Ca^2+^ sensor for endocytosis and functions to couple and balance endocytosis and exocytosis in the vesicle cycle. It has been proposed that a modulatory protein, such as DGKθ, could bind Syt1 during resting states and is released when Syt1 binds Ca^2+^, freeing Syt1 to regulate endocytosis (Yao et al., 2012; Li et al., 2017). It is also possible that the interaction between DGKθ and Syt1 is crucial to coordinate their activities following depolarization. For example, the binding of Syt1 to DGKθ and the subsequent increase of DGKθ’s kinase activity could produce PtdOH that greatly contributes to the negative membrane curvature necessary for vesicle fusion. It is tempting to speculate that Syt1 is a crucial interactor and activator of DGKθ, and together their interaction is important to maintain efficient Ca^2+^-mediated endocytosis (Figure 6). Given Syt1’s indispensable role in vesicle recycling, and recent studies that implicate Syt1 in neurodegenerative disease (Brinkmalm et al., 2014; Gautam et al., 2015; Shi et al., 2020), this previously unknown interaction between DGKθ and Syt1 strengthens the notion that lipid-metabolizing enzymes are important regulators of neurotransmission.

While Syt1 is an attractive candidate for the endogenous DGKθ activator, other proteins, such as syntaxin-7, may also be involved in regulating the enzyme’s activity in neurons. Syntaxin-7 is a SNARE protein mainly localized on early endosomes that is thought to be involved in the homotypic fusion of endocytic organelles and late endosome-lysosome fusion (Prekeris et al., 1999; Ward et al., 2000). As endosomes participate in the replenishment of synaptic vesicles during neuronal activity, it is also important to note that syntaxin-7 mediates endosomal membrane fusion in the rapid reproduction of vesicles during high-frequency repetitive stimulation in hippocampal neurons (Mori et al., 2021). Considering syntaxin-7 also greatly increased DGKθ activity, perhaps there is a role for the interaction of DGKθ and syntaxin-7 in bulk membrane endocytosis following periods of high stimulation. Previous work showed that DGKθ KO neurons possess increasingly slower rates of endocytosis with high frequency stimulation (Goldschmidt et al., 2016). It is generally thought that during periods of high stimulation, bulk membrane endocytosis of large endosomes occurs to balance membrane homeostasis with the high rates of exocytosis (Clayton et al., 2008). It is possible that during periods of high frequency stimulation, DGKθ activated by syntaxin-7, contributes PtdOH in the formation of large endosomes during bulk endocytosis. This interaction could implicate DGKθ in bulk endocytosis and the generation of PtdOH in the membrane curvature of large endosome formation.

Synaptogyrin-1 is an integral membrane protein in synaptic vesicles and produced a ∼2.5-fold increase in DGKθ activity (Figure 5). Although its neuronal function is still unclear, it is thought to regulate neurotransmitter release and have a role in synaptic plasticity (Belizaire et al., 2004). Synaptogyrin-1 and synaptophysin KO mice displayed impaired short- and long-term plasticity (Janz et al., 1999), while overexpression of synaptogyrin-1 in PC12 cells caused inhibition of Ca^2+^-dependent exocytosis (Sugita et al., 1999). Synaptogyrin-1 peptides had one of the most obvious differences between depolarized and non-depolarized samples in our mass spectrometry studies. The number of PSMs was extraordinarily high in non-depolarized samples compared to depolarized and compared to other peptides from the entire experiment. Although a link between synaptogyrin-1 and DGKθ function is not immediately clear, some studies have suggested a role for DGKs in synaptic plasticity (Rodriguez de Turco et al., 2001; Clayton et al., 2008; Barber and Raben, 2020). DGKζ directly interacts with PSD-95 in the postsynapse and contributes to dendritic spine maintenance through its production of PtdOH (Kim et al., 2009). DGKζ KO mice exhibit enhanced long-term potentiation (LTP) and attenuated long-term depression (LTD) at pyramidal synapses in the hippocampus (Seo et al., 2012). One could postulate that the production of PtdOH by DGKθ and its interaction with synaptogyrin-1 might influence synaptic plasticity. Similar to its role in synaptic vesicle endocytosis in the presynapse, DGKθ could potentially regulate AMPA and NMDA receptor-containing vesicle recycling at the postsynapse.

CaMKIIα is the most abundant protein in the neuronal postsynapse, but is also abundant in the presynapse as it associates with synaptic vesicles. Because of its ubiquity, CaMKIIα has been implicated in synaptic plasticity, dendritic arbor structure, and density of glutamatergic synapses, among other things (Fink and Meyer, 2002; Miller et al., 2002; Hell, 2014). Given the breadth of its localization and function, it is difficult to speculate a specific role for CaMKIIα and DGKθ’s interaction in the regulation of synaptic vesicle recycling. As with synaptogyrin-1, however, a possible connection between DGKθ and synaptic plasticity cannot be ignored. Future studies should focus on identifying the role of DGKθ in the postsynapse.

DGKθ’s interaction with Hsc70 may affect the role of Hsc70 in uncoating clathrin from endocytosed vesicles (Eisenberg and Greene, 2007; Böcking et al., 2011). Disruption of DGKθ’s interaction with Hsc70, for example by decreased levels or absence of DGKθ, may slow the rates of clathrin uncoating resulting in slower rates of synaptic vesicle endocytosis as previously observed (Goldschmidt et al., 2016). DGKθ could also participate in the Hsc70-binding of free clathrin after it has disassembled from lattices, to prevent aggregation and quick access for recycling for further rounds of endocytosis. Although several biotinylated peptides from clathrin heavy chain 1 were identified in each of our mass spectrometry experiments (Figure 3B), clathrin heavy chain 1 did not coimmunoprecipitate with DGKθ (Figure 4D). The biotinylation we observed could be the result of the close proximity of DGKθ and clathrin heavy chain 1, due to DGKθ’s binding to Hsc70. We cannot rule out the possibility that our APEX2 proximity labeling detects a very transient interaction between DGKθ and clathrin heavy chain 1 which is not detectable by coimmunoprecipitation.

Interestingly, confirmed interactors dynamin-1 and Munc18-1 did not stimulate DGKθ kinase activity. Munc18-1 binds tightly to syntaxin-1 and is required for SNARE-mediated membrane fusion. Mutations in Munc18-1 cause a defect in the stability of the SNARE bundle and priming of synaptic vesicles (Ferenc et al., 2009; Jiao et al., 2018). Dynamin-1 is a GTPase required for functional endocytosis at synapses. It is thought to oligomerize in a circular shape around the neck of endocytic pits and mediate fission of vesicles from the membrane (Sever, 2002; Raimondi et al., 2011). Dynamin-1 KO synapses have significantly increased numbers of clathrin-coated vesicles and pits that remain connected to membranes following an action potential, leading to a dramatic endocytic defect (Sever, 2002; Ferguson et al., 2007). Our mass spectrometry experiments suggest that both dynamin-1 and Munc18-1 have a stronger interaction with DGKθ following depolarization (Figure 3B and Supplementary Figure 1). Given that both proteins are in close proximity to membrane deformations, a role for DGKθ’s interaction should not be discounted. DGKθ may play a non-catalytic role in regulation or activation of dynamin-1 or Munc18-1. We should also note that Basp1, cofilin-1, PKCα, SNAP-25, and synaptojanin-1 were eliminated as potential DGKθ interactors because we could not biochemically validate their biotinylation. This may be due to very transient interactions between each candidate and DGKθ *in vivo.* For example, SNAP-25 may briefly interact with DGKθ because of DGKθ’s interaction with Munc18-1 as they reside in very close proximity.

It is also important to consider the role of DGKθ-induced PtdOH in compensatory endocytosis. Indeed, many DGKθ-interacting proteins’ functions involve membrane curvature. For example, curvature is important for the dynamin-1-mediated membrane scission, as well as for membrane fusion by the SNARE protein syntaxin-7 and Munc18-1. It is interesting, therefore, to speculate that DGKθ is important for the local production of PtdOH and the generation of that membrane curvature.

Together, we validated that DGKθ interacts with important proteins that regulate key steps of vesicle cycling at the presynapse, most notably Syt1. Future work is required to determine how DGKθ interacts with the interacting proteins identified in this study and which interactions are essential for efficient neurotransmitter release. The identification of DGKθ interactors and activators is crucial for understanding the mechanism of DGKθ’s role in the synaptic vesicle cycle and neurotransmission in general.

## Data Availability Statement

The datasets presented in this study can be found in online repositories. The names of the repository/repositories and accession number(s) can be found in the article/Supplementary Material.

## Ethics Statement

The animal study was reviewed and approved by the Johns Hopkins University Animal Care and Use Committee.

## Author Contributions

CB, HG, RH, and DR: conceptualization. CB, HG, QM, LD, RC, DR, and RH: methodology. CB: investigation. CB, DR, and LD: writing – original draft. CB, HG, RC, DR, and RH: writing, review, and editing draft. DR: funding acquisition. DR and RH: supervision. All authors contributed to the article and approved the submitted version.

## Conflict of Interest

The authors declare that the research was conducted in the absence of any commercial or financial relationships that could be construed as a potential conflict of interest.

## Publisher’s Note

All claims expressed in this article are solely those of the authors and do not necessarily represent those of their affiliated organizations, or those of the publisher, the editors and the reviewers. Any product that may be evaluated in this article, or claim that may be made by its manufacturer, is not guaranteed or endorsed by the publisher.

## Footnotes

1
www.thermofisher.com

2
www.dr-maisch.com

3
www.matrixscience.com

## Supplementary Material

Supplementary Figure 1 Each of the 13 tables corresponds to a protein on our list of candidate DGKθ interactors. Each table outlines the peptides that were found within that protein by listing the number of the amino acid that has the biotin tyramide tag. The number of PSMs for each peptide is also listed. Peptides in the KCl column were more abundant in depolarized samples. Peptides in the control column were more abundant in the non-depolarized samples. PSMs were combined for peptides for which the biotin tyramide tag was located within three amino acids. Peptides with 3 or less PSMs were eliminated from this analysis. Blue text = experiment 1, black text = experiment 2, red text = experiment 3.

Supplementary Figure 2 Basp1, cofilin-1, PKCα, SNAP-25, and synaptojanin-1 were not detected in our streptavidin pulldown experiments and therefore we were not able to confirm biotinylation biochemically for these proteins. They were eliminated from further analysis. Each blot is representative of at least 3 separate experiments.

Supplementary Figure 3 Dynamin-1, synaptogyrin-1, Syt1, CaMKIIα, Munc18-1, Hsc70, and syntaxin-7, all biochemically-confirmed DGKθ-interacting proteins, did not pull down with DGKθ when isolated with a non-specific mouse IgG.

Supplementary Table 1 Detailed data from mass spectrometry experiment 1.

Supplementary Table 2 Detailed data from mass spectrometry experiment 2.

Supplementary Table 3 Detailed data from mass spectrometry experiment 3.
