# Supplementary material for: Identification of Synaptic DGKθ Interactors That Stimulate DGKθ Activity
Source: Front Synaptic Neurosci. 2022 Apr 27;14:855673. doi: 10.3389/fnsyn.2022.855673 (PMC9095502; doi:10.3389/fnsyn.2022.855673)
Supplement: Supplementary Figure 1 — Each of the 13 tables corresponds to a protein on our list of candidate DGKθ interactors. Each table outlines the peptides that were found within that protein by listing the number of the amino acid that has the biotin tyramide tag. The number of PSMs for each peptide is also listed. Peptides in the KCl column were more abundant in depolarized samples. Peptides in the control column were more abundant in the non-depolarized samples. PSMs were combined for peptides for which the biotin tyramide tag was located within three amino acids. Peptides with 3 or less PSMs were eliminated from this analysis. Blue text = experiment 1, black text = experiment 2, red text = experiment 3. [file Image_1.pdf]

Clathrin heavy chain 1			
Control		KCl	
BT Location	#PSMs	BT Location	#PSMs
882	90	626	11
896	26	1095	16
1435	20	1622	10
1482	23		
882	52	626	6
896	15	1095	10
		1435	22
		1482	16
882	42	501	6
		626	10
		1095	28
		1435	22
		1482	45

Dynamin-1			
Control		KCl	
BT Location	#PSMs	BT Location	#PSMs
272	18	310	12
344	33	344	85
386	11		
446	17		
623	38		
272	12	310	9
310	4	344	51
344	4	387	9
386	7	446	13
623	21		
272	7	272	4
344	10	344	85
		367	13
		446	9
		563	5
		623	22

Brain acid soluble protein 1			
Control		KCl	
BT Location	#PSMs	BT Location	#PSMs
10	255	10	221
10	176	10	202
10	208	10	168

Synaptogyrin-1			
Control		KCl	
BT Location	#PSMs	BT Location	#PSMs
1	273		
11	7		
1	177	1	8
11	4		
1	199	1	4

CaMKIIα			
Control		KCl	
BT Location	#PSMs	BT Location	#PSMs
227	36	33	33
434	26	221	84
227	8	33	26
		221	53
		434	19
		33	42
		221	111
		227	63
		434	35

Snap25			
Control		KCl	
BT Location	#PSMs	BT Location	#PSMs
97	164	97	66
		97	184
97	231		

Synaptojanin-1			
Control		KCl	
BT Location	#PSMs	BT Location	#PSMs
439	4	1172	30
		191	4
		1172	21
439	6	191	9
		1172	41

Heat shock cognate 71 (Hsc70)			
Control		KCl	
BT Location	#PSMs	BT Location	#PSMs
138	6	424	4
103	17	362	10
		610	66
		540	6
		518	77
		525	118
		37	6
518	35	525	45
103	6	540	6
610	25	518	55
540	16		
138	7		
138	6	525	74
424	7	518	24
610	4	610	13
540	35		
518	46		
103	9		
129	4		

Cofilin-1			
Control		KCl	
BT Location	#PSMs	BT Location	#PSMs
127	4	133	82
133	7	133	62
133	6	133	79

Syntaxin-binding protein-1			
Control		KCl	
BT Location	#PSMs	BT Location	#PSMs
334	7	468	68
357	45	527	76
466	18		
519	20		
527	35		
334	14	466	53
357	15	519	6
466	14	527	94
498	8		
266	6	357	41
334	9	468	80
468	7	519	12
527	54	527	95

Synaptotagmin-1			
Control		KCl	
BT Location	#PSMs	BT Location	#PSMs
201	5		
214	30		
376	12		
		214	22
		376	5
		214	96
		376	21

Protein Kinase C α			
Control		KCl	
BT Location	#PSMs	BT Location	#PSMs
2	71		
497	13		
2	46		
497	4		
2	52		
497	26		

Syntaxin-7			
Control		KCl	
BT Location	#PSMs	BT Location	#PSMs
2	99	2	7
50	37	50	19
227	8		
2	131	50	58
		2	55
		50	62
		227	10

Supplemental Figure 1.
