# Supplementary figures and images for: Identification of Synaptic DGKθ Interactors That Stimulate DGKθ Activity
Source: Front Synaptic Neurosci. 2022 Apr 27;14:855673. doi: 10.3389/fnsyn.2022.855673 (PMC9095502; doi:10.3389/fnsyn.2022.855673)

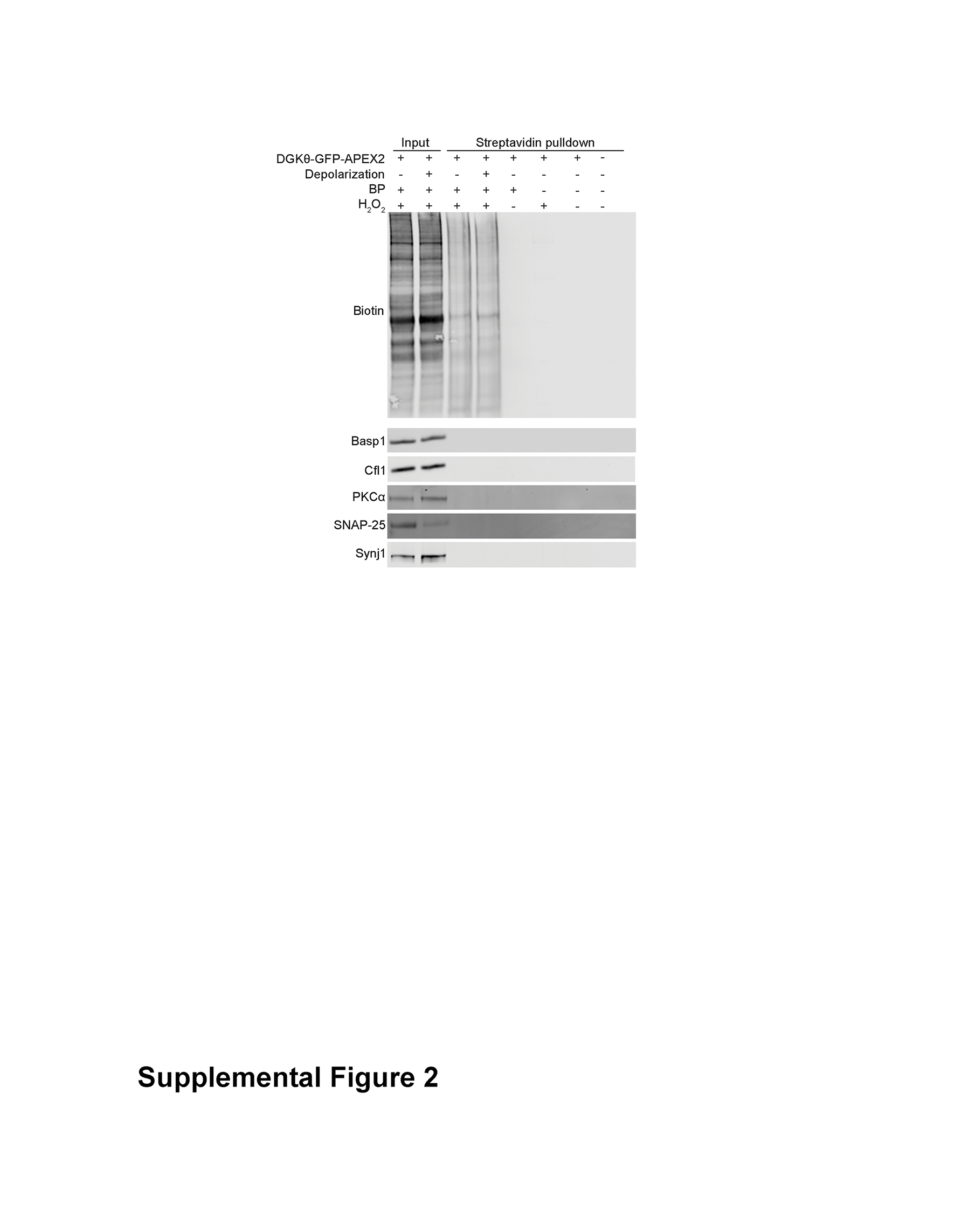

Supplement: Supplementary Figure 2 — Basp1, cofilin-1, PKCα, SNAP-25, and synaptojanin-1 were not detected in our streptavidin pulldown experiments and therefore we were not able to confirm biotinylation biochemically for these proteins. They were eliminated from further analysis. Each blot is representative of at least 3 separate experiments. [file Image_2.tiff]

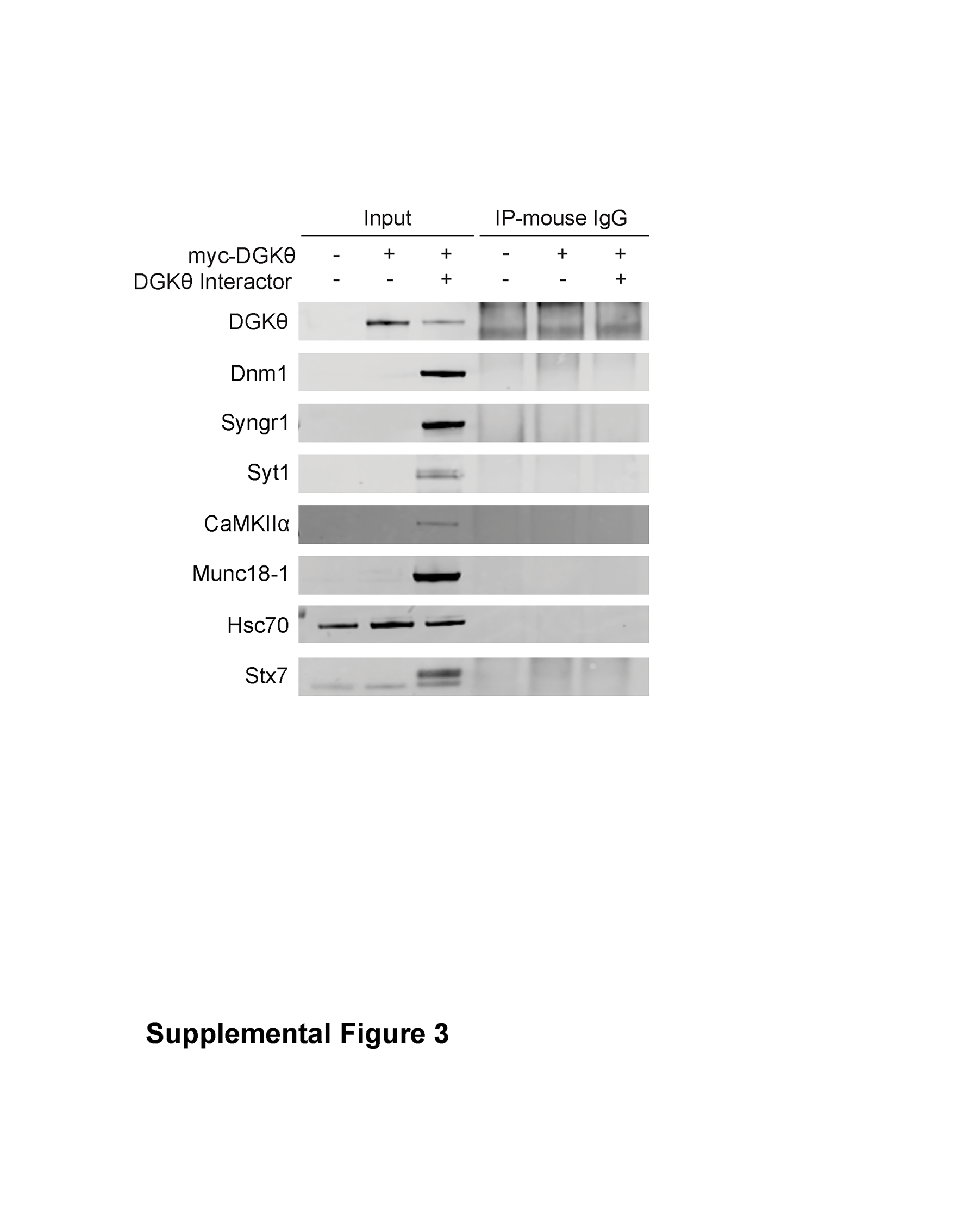

Supplement: Supplementary Figure 3 — Dynamin-1, synaptogyrin-1, Syt1, CaMKIIα, Munc18-1, Hsc70, and syntaxin-7, all biochemically-confirmed DGKθ-interacting proteins, did not pull down with DGKθ when isolated with a non-specific mouse IgG. [file Image_3.tiff]
